# Robust Medical Image Colorization with Spatial Mask-Guided Generative Adversarial Network

**DOI:** 10.3390/bioengineering9120721

**Published:** 2022-11-22

**Authors:** Zuyu Zhang, Yan Li, Byeong-Seok Shin

**Affiliations:** Department of Electrical and Computer Engineering, Inha University, Incheon 22212, Republic of Korea

**Keywords:** image colorization, generative adversarial network, medical images

## Abstract

Color medical images provide better visualization and diagnostic information for doctors during clinical procedures than grayscale medical images. Although generative adversarial network-based image colorization approaches have shown promising results, in these methods, adversarial training is applied to the whole image without considering the appearance conflicts between the foreground objects and the background contents, resulting in generating various artifacts. To remedy this issue, we propose a fully automatic spatial mask-guided colorization with generative adversarial network (SMCGAN) framework for medical image colorization. It generates colorized images with fewer artifacts by introducing spatial masks, which encourage the network to focus on the colorization of the foreground regions instead of the whole image. Specifically, we propose a novel spatial mask-guided method by introducing an auxiliary foreground segmentation branch combined with the main colorization branch to obtain the spatial masks. The spatial masks are then used to generate masked colorized images where most background contents are filtered out. Moreover, two discriminators are utilized for the generated colorized images and masked generated colorized images, respectively, to assist the model in focusing on the colorization of foreground regions. We validate our proposed framework on two publicly available datasets, including the Visible Human Project (VHP) dataset and the prostate dataset from NCI-ISBI 2013 challenge. The experimental results demonstrate that SMCGAN outperforms the state-of-the-art GAN-based image colorization approaches with an average improvement of 8.48% in the PSNR metric. The proposed SMCGAN can also generate colorized medical images with fewer artifacts.

## 1. Introduction

Imaging technology in biomedical engineering has made the interior portions of the body observable by doctors for disease diagnoses without having to invade the bodies of patients [1,2]. Medical imaging has also been used to guide and assist surgical procedures [3]. For instance, in keyhole surgeries, can help doctors reach the interior parts without really opening too much of the body. Medical imaging utilizes fundamental physical phenomena, including acoustic wave dissemination and X-ray propagation, to obtain the health parameters of patients. With the emergence of advanced medical imaging devices, a large number of medical images have been generated and collected at an unprecedented speed and scale using imaging techniques such as computed tomography (CT), magnetic resonance imaging (MRI), and ultrasound imaging (UI) [4]. Thus, it is critical to develop medical image processing algorithms to help doctors diagnose and analyze diseases rapidly. Among them, medical image colorization is an important topic attracting more and more attention [5]. Synthesized color images can enrich the details of organs and tissues much more compared with gray images [6,7]. They can help doctors identify problems more accurately and avoid misjudgment [8].

Image colorization is the process of assigning colors to each pixel based on the intensity in a grayscale image. In the last decade, many methods have been proposed to solve this problem. These algorithms can be roughly divided into three categories: scribble-based methods [9,10], exemplar-based methods [11,12], and fully automatic methods [13,14,15,16]. The scribble-based methods utilize the color hints provided by users to assign different colors to the objects in an image. In contrast, to reduce human interactions during the process of colorization, exemplar-based methods infer the RGB color of each region in the input image by selecting a reference image. However, the performance of example-based methods depends highly on the quality of the selected reference image.

Different from scribble-based methods and exemplar-based methods, fully automatic approaches based on deep learning perform colorization in an end-to-end manner without any human intervention [15,16]. Recently, GAN-based colorization methods have been explored to perform fully automatic colorization. For example, Nazeri et al. proposed the deep convolutional generative adversarial network (DCGAN) [15] to perform end-to-end colorization by directly learning the mapping between the input grayscale images and the corresponding colorized ones. Vitoria et al. [16] proposed ChromaGAN, an adversarial learning colorization approach with semantic information incorporated into it to colorize images more realistically. Although the aforementioned GAN-based methods have achieved promising results, these GAN-based methods mainly focus on adversarial training operated on the whole image while neglecting the various generated artifacts [17,18]. Some artifacts are produced because of appearance conflicts between the foreground objects and the background contents [19]. In medical images, background regions may not contain any tissues or organs and can be regarded as noise during the colorization process. Several works have been proposed to employ foreground-aware adversarial training to suppress the generated artifacts [19,20]. However, the effectiveness of foreground-aware adversarial training has not been investigated in image colorization for medical images. In addition, unlike most previous works that were limited to natural image colorization, this work focuses on the colorization techniques of medical images.

To this end, we propose a novel end-to-end SMCGAN framework for medical image colorization by introducing a spatial mask-guided generative adversarial network, in which the model is forced to focus on the colorization of foreground regions and reduce visual artifacts. Specifically, we employ a generative adversarial network as the main image colorization network to learn the mapping between the grayscale values and chromatic values. A spatial mask derived from the auxiliary segmentation network is used to obtain a weighted synthesized color image where most contents of background regions are filtered out. The weighted synthesized color image assists the image colorization network to focus the colorization of the foreground regions by adversarial training.

The main contributions of this paper are as follows:

- We demonstrate that the foreground-aware module composed of a spatial mask embedded in a GAN-based colorization framework makes the model emphasize the foreground regions during the colorization process.
- A novel adversarial loss function is devised to assist the colorization model to focus on the colorization of foreground regions, reducing visual artifacts to improve the performance of colorization.

The remainder of this paper is organized as follows: we introduce the existing methods of image colorization in Section 2. In Section 3, we detail the architecture and the loss functions of our proposed SMCGAN framework. Performance comparisons and ablation studies of the proposed SMCGAN framework are performed in Section 4. We also provide a brief analysis of the limitations of our framework in Section 5. Finally, in Section 6, we present our conclusions.

## 2. Related Work

The existing methods of image colorization can be divided into three categories: scribble-based colorization, exemplar-based colorization, and fully automatic-based colorization.

Scribble-based methods attempt to annotate the grayscale image in a straightforward way with color scribbles. These color scribbles serve as landmarks for colorization, and color from the scribble is propagated to the rest of the image. Levin et al. [9] regarded scribble-based colorization as an optimization problem with linear constraints based on the assumption that the adjacent pixels with the same intensity should have a similar color. To capture the long-range color relationships, both the use of locally linear embedding [21] and the utilization of an affinity-based edit scheme [22] are proposed by modeling the linear combination of adjacent pixels in a featured space. To maintain structural information, Sangkloy et al. [23] used a novel deep generative adversarial architecture with sketches and color strokes as user input. To simultaneously utilize global and local information, Xiao et al. [24] developed an interactive colorization model based on the U-Net [25] architecture, which is composed of a feature extraction module, a dilated module, a global input module, and a reconstruction module. However, the main weakness of the aforementioned scribble-based methods is that the results are highly related to the position and number of given color scribbles. In addition, most of these methods demand significant human efforts to provide hints for ensuring plausible colorization results.

In contrast, the exemplar-based colorization approaches exploit color information from a referenced source image to guide the colorization of the target grayscale image. They reduce human effort in choosing many color scribbles and mainly focus on matching local spatial features between the reference image and the input grayscale image by using statistical analysis [26]. Recently, deep learning techniques have been employed in the exemplar-based colorization methods to further reduce human intervention during image colorization. He et al. [27] proposed the first deep exemplar-based method to transfer the colors from a reference image to the grayscale one, where the network is composed of a similarity subnetwork for automatically recommending references and a colorization subnetwork for colorizing images. A faster version of deep exemplar colorization is proposed by Xu et al. [28] with a stylization-based architecture. To generate semantically related colorized images from reference images, both Gray2ColorNet [29] with an attention-gating mechanism-based color fusion network and reference-based sketch image colorization [30] with augmented-self reference are proposed. However, the results generated by these exemplar-based methods are highly dependent on the quality of the reference images. In other words, unnatural output images would be obtained when a given reference image exhibits a large variance from the input image.

Recently, various deep learning-based models have been proposed for full automatic colorization without any human efforts [31,32,33]. Most early works employ a simple, straightforward architecture with stacked convolutional layers to learn the mapping from grayscale to color embeddings [34]. For example, Cheng et al. [31] first introduced deep neural networks to implement image colorization by learning a mapping function between features extracted from patches in a grayscale image and color values of the source image. An et al. [35] developed a fully automatic learning-based colorization algorithm on the VGG-16 CNN model based on the classification with the loss of cross-entropy. To extract features at different levels, multi-path networks [32,36] have been proposed. For instance, to preserve global features during model training, Iizuka et al. [32] developed two-stream networks to extract local features and global features, respectively. A fusion layer was utilized to fuse local and global information together. However, these CNN-based image colorization methods may produce blurry results because the Euclidean distance is minimized by averaging all plausible outputs [37]. More recently, to produce vivid colorization results, some generative models have been proposed for image colorization [13,16,38]. Isola et al. [13] proposed a general image-to-image translation framework based on conditional GAN to produce plausible results by adversarial training. Vitoria et al. [16] proposed ChromaGAN to colorize by combining both the perceptual and semantic understanding of color and class distributions in an adversarial training manner. To minimize semantic confusion and color bleeding, Zhao et al. [39] proposed a fully automatic saliency map-guided colorization with a generative adversarial network (SCGAN) framework, which jointly predicts the colorization and saliency map. However, most of these GAN-based image colorization approaches emphasize adversarial training to mimic the distribution of real color images while various generated artifacts are neglected. The proposed SMCGAN framework produces high-quality colorized images while reducing the generated artifacts resulting from various backgrounds.

## 3. Methodology

**Figure 1:**
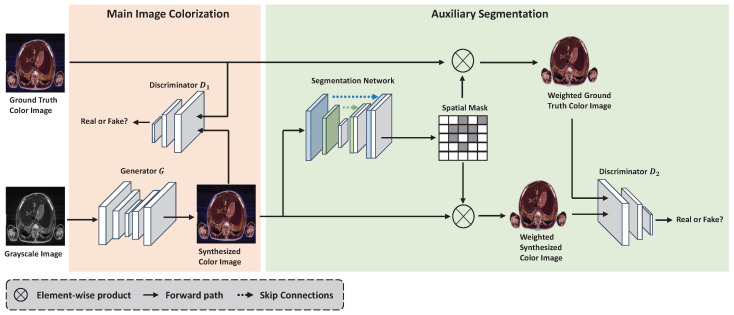
Overview of the proposed SMCGAN. It receives a grayscale image as input and predicts a colorized image and a corresponding spatial mask. It comprises a main colorization network and an auxiliary segmentation network.

Image colorization is an image-to-image translation problem that maps a grayscale image to a color image. In this work, we follow the work of Nazeri et al. [15] and utilize the YUV color space for the colorization task. Thus, image colorization can also be seen as a pixel-wise regression problem that maps the grayscale value of each pixel to the chromatic value of the corresponding pixel. Different from the GAN-based image colorization methods focusing on adversarial training [31], our work aims to reduce the generated artifacts caused by the various backgrounds when learning the mapping between grayscale values and chromatic values. Motivated by the work of Wang et al. [40], we introduce spatial masks in our proposed SMCGAN framework to identify foreground organs and tissues, as illustrated in Figure 1. The work in [40] aims to prune redundant computation in flat regions for CNN-based super-resolution by using spatial masks to identify important regions in feature maps. While our proposed method aims to learn robust colorization for medical images by using spatial masks on the synthesized color images. In this way, during the process of color transfer, the image colorization model is forced to focus on the foreground regions containing organs or tissues rather than on the background regions.

### 3.1. SMCGAN Architecture

An overview of the SMCGAN framework is shown in Figure 1. Our method is extended from GAN architecture [41] that simultaneously produces colorized images and spatial masks from grayscale images. It is composed of a main colorization network and an auxiliary segmentation network. As we regard image colorization as image-to-image translation, we employ an image transformation network first proposed by Johnson et al. [42] as the main colorization network and the generator *G* to colorize the input grayscale images. It comprises five residual blocks with non-residual convolutional layers followed by batch normalization and ReLU nonlinearities with the exception of the output layer. We employ the plain U-Net structure [25] as the auxiliary segmentation network. U-Net employs skip connections, which connect the output feature map generated from each level of the encoder to the corresponding level of the decoder. These skip structures have been proven to be effective in preventing gradient vanishment and simultaneously fusing low-level features and global features [43]. Both low-level features and global features play an important role in medical image segmentation. We also follow Dong et al. and Lee et al. [44,45] and share the same ResNet18 architecture [46] for the two discriminators. ResNet18 is widely used as a discriminator to distinguish images due to its strong feature extraction ability, which is composed of residual building blocks. The first discriminator, $D_{1}$, judges the synthesized color image and ground truth color image. A weighted colorized image is obtained by performing an element-wise product between the synthesized color image and the generated spatial mask. Similarly, a weighted ground truth color image can also be obtained by performing an element-wise product between the ground truth color image and the generated spatial mask. Thereafter, we feed the paired weighted color images to the second discriminator, $D_{2}$, which judges whether the input is real weighted color images or not. For example, given a grayscale medical image $x^{y}$, the main colorization network first translates the grayscale medical image into a colorized image $G(x^{y})$ that cannot be distinguished from the ground truth color image *x*. The discriminator $D_{1}$ is then trained to distinguish between the synthesized color image and the real color image well. The spatial mask is obtained from the auxiliary foreground segmentation task and utilized to produce the weighted ground truth color image and the weighted synthesized color image. Finally, discriminator $D_{2}$ is utilized to distinguish between the weighted ground truth color image and the weighted synthesized color image. Regarding the loss functions, a segmentation loss based on cross-entropy, an adversarial loss, and a color loss based on an L1 term is defined. We will detail each loss term in the next section.

To reduce the impact of the background when performing colorization, we introduce a spatial mask embedding in the GAN-based image colorization structure to identify the foreground regions containing organs and tissues. The spatial mask is used to act on the synthesized color image to obtain a weighted synthesized color image where most background contents are masked. A spatial mask is defined to be a binary matrix where only the pixels within the bounding box area are nonzero [47]. The spatial mask $M_{s}$ has the same spatial size as the synthesized color image $\hat{x}$, which can be written as follows:

(1) $$M_{s}\left(i,j\right)=\left\{\begin{array}{cc} 1, & S[\hat{x}\left(i,j\right)]\geq 0.5\\ 0, & \;\mathrm{Otherwise}\; \end{array}\right.$$

where *S* denotes the foreground segmentation network, and *i* and *j* represent the vertical and horizontal indices of an image, respectively. The term $S[\hat{x}\left(i,j\right)]$ means the output of the foreground segmentation network using the synthesized color image $\hat{x}$ as the input.

However, during training, there is a challenge that the binary spatial mask cannot directly backpropagate, as it is non-differentiable. Here, we utilize the Gumbel-softmax reparameterization technique [48,49] to relax the discrete binary masks to continuous variables. Specifically, the probability of the foreground regions being selected is $P_{s}^{1}=S\left[\hat{x}\left(i,j\right)\right]$. In contrast, the probability of the background regions being selected is $P_{s}^{0}=1-S\left[\hat{x}\left(i,j\right)\right]$. Then, the sampling process of the spatial mask $M_{s}$ can be reparameterized as:

(2) $$M_{s}=\underset{k}{\arg\max}\left(\log\left(P_{s}^{k}\right)+g_{k}\right),\forall k=0,1$$

where ${\left\{g_{k}\right\}}_{k=\{0,1\}}$ are random variables that follow the Gumbel distribution. To make the spatial mask $M_{s}$ continuous, we replace the discontinuous function argmax with a softmax. Then, the binary learnable spatial mask from the Gumbel-softmax relaxation can be expressed as follows:

(3) $$M_{s}=\frac{\exp\left(\frac{\log\left(P_{s}^{1}\right)+g_{1}}{\tau }\right)}{\Sigma_{k\in \{0,1\}}\exp\left(\frac{\log\left(P_{s}^{k}\right)+g_{k}}{\tau }\right)}$$

where $\tau \in \left(0,\infty \right)$ is a temperature parameter. The Gumbel-softmax distribution becomes a uniform distribution when τ→∞. Conversely, samples from the Gumbel-softmax distribution become one-hot. In other words, $M_{s}$ becomes a binary mask. In the experiments, we empirically start τ with a high temperature and gradually decrease it to a lower value.

To emphasize the foreground areas where organs or tissues are located, we perform element-wise product between synthesized color images and the spatial masks generated from the foreground segmentation network. The weighted output synthesized images contain regions filtering out most background contents and are sent to the discriminator for adversarial training. In this way, the foreground segmentation network is capable of assisting the main colorization network to focus on the colorization of the foreground regions instead of the background regions.

### 3.2. Loss Functions

Here, $x^{y}$ denotes the luminance (Y) of the input image *x* on the YUV color space, while $x^{uv}$ represents the chrominance (UV). We propose a color loss based on the L1 loss to guide the color of the synthesized image.

(4) $$L_{col}=E[∥G\left(x^{y}\right)-x^{uv}{∥}_{1}+∥G\left(x^{y}\right)⊙M_{s}-x^{uv}⊙M_{s}{∥}_{1}],$$

where the operator ⊙ means the element-wise product and ${∥\cdot ∥}_{1}$ denotes the L1 norm. The color loss makes the colorization model learn the mapping between grayscale values and chromatic values. To generate target-like colorized images from the luminance of the input grayscale image, we also propose two additional adversarial losses derived from two discriminators, $D_{1}\left(\cdot \right)$ and $D_{2}\left(\cdot \right)$, respectively. The proposed adversarial loss items are defined as:

(5) $$\begin{array}{cc} L_{adv} & =L_{gan1}+L_{gan2}, \end{array}$$

(6) $$\begin{array}{cc} L_{gan1} & =E[\log D_{1}\left(x\right)+E\left[\log\left(1-D_{1}\left(fc\left(G\left(x^{y}\right),x^{y}\right)\right)\right)\right], \end{array}$$

(7) $$\begin{array}{cc} L_{gan2} & =E[\log D_{2}\left(x⊙M_{s}\right)+E\left[\log\left(1-D_{2}\left(fc\left(G\left(x^{y}\right),x^{y}\right)⊙M_{s}\right)\right)\right], \end{array}$$

where *D* denotes the discriminator of the networks and $f_{c}\left(\cdot \right)$ is a concatenation function used to concatenate the luminance and chrominance to regenerate an image. $L_{gan1}$ means the adversarial loss between the ground truth color image and the synthesized color image. $L_{gan2}$ represents the adversarial loss between the weighted ground truth color image and the weighted synthesized color image.

In the task of foreground segmentation, we adopt U-Net [25] to perform foreground segmentation with the cross-entropy loss. The spatial masks are derived from the auxiliary segmentation network. Although the learned foreground maps are not accurate during the first several iterations, the synthesized colorized medical images can still reduce visual artifacts by combining both adversarial losses and cross-entropy loss after adequate iterations. The segmentation loss can be written as:

(8) $$L_{seg}=E\left[m\left[-\log\left(p\right)\right]+\left(1-m\right)\left[-\log\left(1-p\right)\right]\right],$$

where $p=S(fc\left(G\left(x^{y}\right),x^{y}\right))$ is the output of the foreground segmentation, and *m* denotes the ground truth map of foreground segmentation (i.e., 0 for background regions and 1 for foreground regions).

By combining all the aforementioned losses, the total loss function of the proposed SMCGAN framework can be described as follows:

(9) $$L_{total}=L_{adv}+\lambda_{col}L_{col}+\lambda_{seg}L_{seg},$$

where $\lambda_{col}$ and $\lambda_{seg}$ are the weight parameters of the color loss function and segmentation loss function, respectively.

## 4. Experiments and Analysis

### 4.1. Experimental Settings

For datasets, we adopt the publicly available Visible Human Project (VHP) [50] dataset to evaluate our methodology. This dataset provides cross-sectional cryosection, MRI, and CT images of two cadavers, including one male cadaver and one female cadaver. We also adopt prostate T2-weighted MRI images from the NCI-ISBI 2013 challenge [51] for evaluation. This dataset contains 30 samples (578 images) collected by Radboud University Nijmegen Medical Centre with a resolution of in-plane of 0.6–0.625 mm and through-plane of 3.6–4.0 mm. For the cross-sectional cryosection images of VHP, only the thorax images and the abdomen images are selected for colorization performance evaluation, as these images contain a number of organs and tissues. The foreground masks of the cryosection images are first obtained by feeding into the pre-trained salient object detection model [52] to reduce the workload of annotations and then refined by professional doctors. We train the model on the cross-sectional cryosection datasets with original backgrounds and test it on the cross-sectional cryosection datasets with different backgrounds and the remaining datasets. All the images are rescaled to 256×256 and normalized within a [−1, 1] range. For quantitative metrics, we adopt pixel-level MAE (mean absolute error) to evaluate the prediction accuracy of the synthesized color images, and PSNR (peak signal to noise ratio) and SSIM (structural similarity index) metrics to evaluate the pixel fidelity of an image [15,39,53]. For perceptual evaluation, we adopt color naturalness and color bleeding removal [39]. The color naturalness denotes whether the color of the colorized images is reasonable. For instance, the color of the same tissues should be the same. Different from color naturalness, color bleeding artifacts exist around the region boundaries of colorized images. A robust colorization system is capable of reducing such artifacts. For comparison methods, we adopt six GAN-based fully automatic colorization methods, including GAN [41], DCGAN [15], ChromaGAN [16], WGAN [54], WGAN-GP [55], and CycleGAN [56], for comparisons.

### 4.2. Implementation Details

For datasets, the ground truth foreground segmentation maps are generated by a pre-trained BASNet [52]. The cross-sectional cryosection images with different backgrounds are obtained by replacing their backgrounds. For network architecture, we employ the image transformation network in [42] as the generator (main colorization network), the ResNet18 architecture [46] as the two discriminators, and the U-Net structure [25] as the auxiliary segmentation network. For optimization details, we train the generator, segmentation network, and discriminators collaboratively for 10 epochs. We use the Adam optimizer with a batch size of 1 and a learning rate of $1\times 10^{-4}$. The trade-off parameters $\lambda_{col}$ and $\lambda_{seg}$ are empirically set to 1.0 and 1.0, respectively. We start the temperature τ at 1.0 and decrease it by $1.0-t/t_{max}$, where *t* and $t_{max}$ denote the current and maximum training epochs, respectively. For quantitative tasks, we repeat the experiments three times and report the average performance. We implement the whole framework in Pytorch framework, using a single NVIDIA 1080Ti GPU.

### 4.3. Experimental Results

**Comparison with state-of-the-art methods.** The comparison colorization results using cross-sectional cryosection images with different backgrounds between SMCGAN and the other GAN-based colorization methods are shown in Table 1. From these results, we can observe that our proposed SMCGAN outperforms the other colorization methods in terms of MAE, achieving an average MAE of 0.019. It means that the proposed method can accurately learn the mapping between grayscale values and chromatic values. In addition, our proposed SMCGAN also ranks first in the PSNR metric as well as the SSIM metric, achieving an average PSNR of 27.42 and an average SSIM of 0.983. It demonstrates that our proposed SMCGAN could accurately model the perceptual structure of grayscale input images and also generate colorized images of good quality. DCGAN performs better than GAN in terms of MAE, with an improvement of 0.040. WGAN and WGAN-GP perform better than both GAN and DCGAN in terms of the MAE, PSNR, and SSIM metrics as they mitigate the issue of unstable training and synthesize images with better quality. CycleGAN obtains similar performance to WGAN and WGAN-GP as the cycle consistency is utilized to generate images with content information reserved. ChromaGAN performs better than the other baseline methods in terms of PSNR metric, as the semantic information is considered when performing colorization. This indicates that a good semantic understanding of color can help the model accurately learn the mapping function for colorization. The proposed SMCGAN can be considered robust because even compared with ChromaGAN, it still has an average improvement of 0.011 and 1.17 over the MAE and PSNR metrics, respectively. This demonstrates that our method is capable of being more accurate and obtaining more robust colorization results than the other comparative methods.

**Figure 2:**
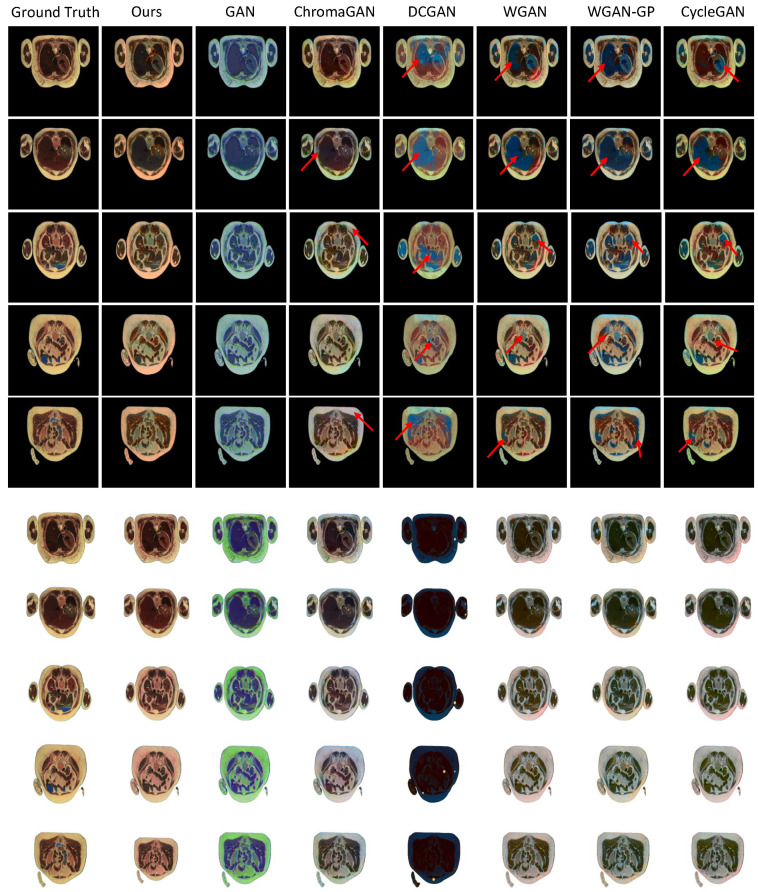
Qualitative colorization results over the cross-sectional cryosection images with black background images and white background images, respectively. The first five rows show the colorization results using images with black backgrounds as input, while the last five rows are the colorization results using images with white backgrounds. The red arrow denotes the artifacts of the synthetic colorized images.

The visualization colorization results of the proposed SMCGAN and the other methods using cross-sectional cryosection images with different backgrounds are illustrated in Figure 2 for qualitative evaluation. The results from GAN and DCGAN between the two backgrounds are inconsistent in chromatic value. This demonstrates that the background of color images has a significant impact on the colorization process for GAN-based colorization algorithms. As GAN-based colorization approaches aim to synthesize the whole colorized images that are indistinguishable from the real colorized images, the perturbation in the background would lead to generating inconsistent results. Moreover, the results from GAN and DCGAN between two backgrounds exhibit a large shift in chromatic value compared with the ground truth color images. This shows that neither GAN nor DCGAN is capable of accurately learning the mapping between grayscale values and chromatic values. This inaccurate mapping between grayscale values and chromatic values also exists in WGAN, WGAN-GP, and CycleGAN. In contrast, the synthesized color images from ChromaGAN are similar to the ground truth color images. This implies that the incorporation of semantic information results in improving the ability to learn the mapping function for colorization, even if some perturbations exist in the background of color images. Compared with ChromaGAN, our proposed SMCGAN produces more similar colorized images to the ground truth color images. Although semantic information is not utilized in our proposed SMCGAN, it is still able to accurately learn the mapping between grayscale values and chromatic values. This demonstrates the utility of introducing spatial masks, which force the colorization model to emphasize the foreground regions and improve the performance of image colorization.

**Figure 3:**
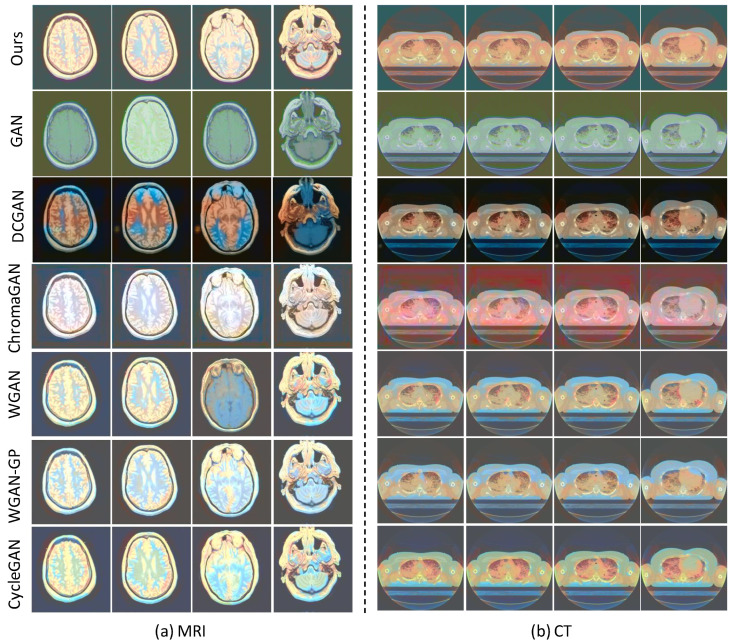
Qualitative colorization results of MRI images and CT images of the VHP dataset using different GAN-based image colorization methods.

**Figure 4:**
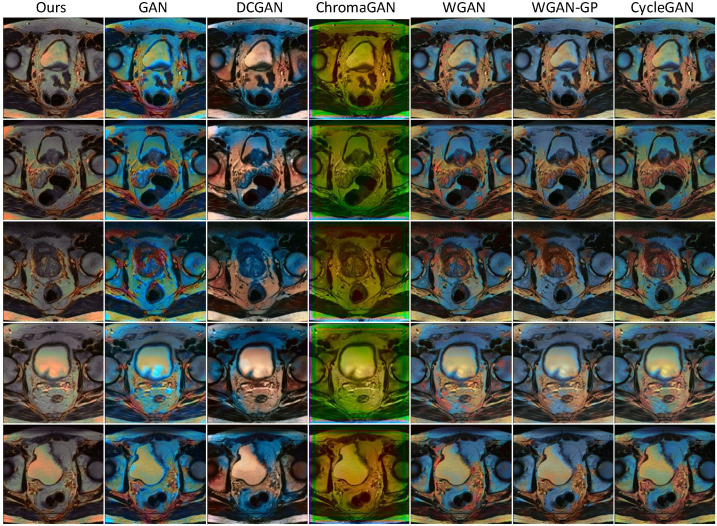
Qualitative colorization results of the prostate T2-weighted MRI using different image colorization approaches.

To evaluate the generalization capability of the proposed SMCGAN in colorization qualitatively, we also produce visualization colorization results of different GAN-based image colorization methods using MRI and CT datasets, as illustrated in Figure 3 and Figure 4. It is to be noted that ground truth color images are not available in these datasets. Consequently, we evaluated the performance through a perceptual analysis, including color naturalness and color bleeding removal. From the results in Figure 3a, we can observe that the color of the colorized images generated by GAN are almost the same. It is not reasonable because different types of tissues should be rendered in different colors. This indicates that GAN fails to learn the mapping between different grayscale values and different chromatic values. In contrast, different tissues of colorized images in DCGAN are rendered in different colors. However, some color-bleeding artifacts exist between the borders of different tissues. For instance, the colors of some tissues bleed into the other tissues, as seen in the first column image of DCGAN. This shows that DCGAN fails to capture the semantic information when performing image colorization. Similar artifacts also exist in WGAN, WGAN-GP, and CycleGAN. The colorized images generated by ChromaGAN contain fewer artifacts than the other baseline methods. As ChromaGAN considers semantic information during image colorization, it is able to learn the relationship between tissues and colors. Compared with ChromaGAN, the colorized images generated by our proposed SMCGAN are more reasonable and natural as the colors are diverse and the structural details of the tissues are clearer. The results in Figure 3b are similar to those in Figure 3a. Both ChromaGAN and our proposed SMCGAN achieve better performance than GAN and DCGAN in terms of color naturalness and color-bleeding removal. As many details are missing in the CT images, it is more practical to compare the MRI images to test the colorization performance. From the image colorization results of prostate T2-weighted MRI in Figure 4, we can observe that the colorized images generated by our approach contain a clearer structure and fewer artifacts compared with those synthesized by the other image colorization algorithms. From these results, we can conclude that our approach is capable of generating robust results and reducing color-bleeding artifacts.

**The effectiveness of using the foreground maps.** Although the generalization ability of our proposed approach has been validated, the effectiveness of using the foreground map is still required to be investigated. Therefore, we perform a comparison between using the coarse foreground maps and refined maps, as shown in Table 2. Note that the coarse foreground maps directly obtained from the pre-trained salient object detection model [52] contain many noise labels, while the refined maps have accurate annotations on the cryosection images. From the results, we can conclude that the performance of the model using the coarse foreground maps decreased significantly as they may mislead the model in learning to distinguish the foreground regions and background regions.

**Ablation analysis of our approach.** To further investigate the effectiveness of several loss functions, we analyzed the different loss functions of our proposed SMCGAN on cross-sectional cryosection dataset quantitatively. Basically, there are three settings to exclude some parts from the original structure: (1) Drop the color loss to investigate the effect of the color loss $L_{col}$. This setting will not affect the network architecture. (2) Drop the discriminators and generators for colorized images, with adversarial training to analyze the effect of the adversarial loss $L_{gan1}$ in SMCGAN. (3) Drop the discriminators and generators for weighted colorized images, with adversarial training to analyze the effect of the adversarial loss $L_{gan2}$ in SMCGAN.

The quantitative analysis of the ablation study is summarized in Table 3. First, if the color loss is removed, the model tends to fail to accurately learn the mapping between the grayscale values and the chromatic values. The performance drops significantly compared with full losses in terms of MAE, PSNR, and SSIM. Secondly, if the gan1 loss is dropped, the performance is still inferior to full losses. The gan1 loss plays a key role in modeling the intensity distribution of colorized image values. Finally, the SMCGAN without gan2 loss produces results that are worse than the SMCGAN without gan1 loss. This result also demonstrates the effectiveness of the spatial mask in the improvement of colorization performance. In conclusion, each component of the proposed SMCGAN is indispensable.

**Figure 5:**
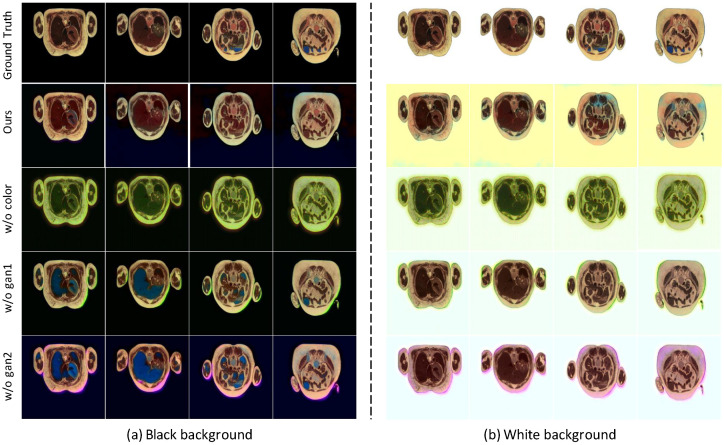
A comparison of colorization results under different ablation study settings. (**a**) Colorization results using images with black background as input; (**b**) Colorization results using images with white background.

As shown in Figure 5, the full SMCGAN produces the best perceptual results compared with the three settings of the ablation study. If the color loss is removed, the color of the generated images exhibits a large discrepancy in chromatil value compared with the ground truth color images. Moreover, the generated images are less colorful than the full SMCGAN. This demonstrates the importance of color loss in learning the mapping between grayscale values and chromatic values. If gan1 loss is reduced, the color of the synthesized images still has less shift in chromatic values than full SMCGAN. If gan2 loss is reduced, the color-bleeding artifacts are generated in the samples. This result also validates the effectiveness of introducing spatial masks to make the model focus on the colorization of foreground regions and reduce generated artifacts.

## 5. Discussion

Although the proposed SMCGAN has achieved robust colorization results over cryosection images, CT images, and MRI images, there are still several issues needed to be further discussed.

**Why the BASNet is utilized to obtain segmentation maps.** Background subtraction (BGS) aims to segment the foreground objects from their surroundings in a given image [57,58,59]. The key issue of BGS is how to improve the accuracy of the detection of the foreground [57]. The Gaussian mixture model (GMM) is one of the widely used models for background modeling [58,59], which attempts to model color intensity variations as a mixture of Gaussians at the pixel level. However, the GMM-based methods are not robust and suffer from performance degradation when they encounter complex scenes. Recently, CNN-based methods have demonstrated significant performances for foreground detection [60,61,62]. However, most of these methods follow the trend of recent generic image segmentation, including multi-scale feature aggregation, concatenated features from different layers, and multi-scale inputs [63]. Different from these aforementioned methods, BASNet pays more attention to the finer structures rather than the large structures [52]. We thus employed a pre-trained BASNet to obtain segmentation maps of the VHP dataset as ground truth, considering small tissues such as blood vessels are required to be treated carefully during the image segmentation process.

**Why not utilize the foreground map as a mask?** To guide the image generation network focusing on the colorization of organs or tissues, we embed the spatial mask obtained from the foreground map into the whole network. As the foreground map is a non-differentiable binary spatial map, we can not directly employ it in the network, considering the backpropagation during the model training process. We thus propose to utilize the Gumbel-softmax reparameterization technique [48,49] to relax the discrete binary masks to continuous variables. In this way, the segmentation subnetwork is capable of assisting the image colorization network in paying more attention to the colorization of foreground organs or tissues instead of the background noises.

**How to avoid miss-coloring of the regions with indistinct border margins?** We have proposed a fully automatic approach utilizing end-to-end learning to directly learn the mapping between an input grayscale image and the corresponding color image without the requirement of user intervention. Like other popular image colorization approaches [64], we also adopt the widely-used L1 loss to regularize the difference between the synthetic color image and the real color image on the chrominance space. Nevertheless, the L1 loss treats the colorization as a regression or classification problem at the pixel level, which may fail in accurately coloring the regions with indistinct border margins. To alleviate this issue, some previous works [32,65] encoded the semantic information to guide image colorization at the image level. However, most of these methods are designed for specific scenes relying on pre-trained models utilized for feature extraction. Different from these methods, we propose to employ an auxiliary segmentation branch with spatial mask to help focus more on the organs or tissues while they are less influenced by the background. In this way, our proposed method is capable of accurately coloring the regions between the foreground objects and the backgrounds, even with indistinct border margins.

**Disadvantages and future works.** Although the proposed SMCGAN can generate relatively robust colorized images in most cases, there are still some failure cases, as shown in Figure 2. The color breeding artifacts still exist in some generated images. As there is no specific loss item for enhancing colors by considering the semantic information of medical images, it is difficult to identify the colors for organs or tissues at the pixel level when the background content changes significantly. Some previous works [53,66] have employed pre-trained VGG models to extract semantic information; these VGG models are trained on natural image datasets, which may not be capable of effectively capturing the semantic features for medical images due to the large discrepancy compared with natural images. In the future, we will develop new methods for medical image colorization while considering semantic information.

## 6. Conclusions

We presented a fully automatic SMCGAN colorization framework for medical images to reduce the generated artifacts. It simultaneously generates colorized images and their corresponding spatial masks from grayscale input images by introducing an auxiliary foreground segmentation network combined with the main colorization network. The generated spatial masks can be used to generate weighted synthesized color images where most background contents are filtered out, which assists the discriminator in emphasizing the colorization of foreground regions and reducing the generated artifacts. We validated our proposed framework on the publicly available VHP dataset and the prostate dataset from NCI-ISBI 2013 challenge, compared with six state-of-the-art GAN-based image colorization. The experimental results demonstrated that SMCGAN can generate robust colorized medical images and reduce generated artifacts.

## Figures and Tables

**Table 1:** Comparison results using different background images as input over cross-sectional cryosection images in terms of the MAE, PSNR, and SSIM metrics. The small *p*-values (*p* < 0.001) calculated between our method and the comparison approaches in terms of MAE indicate the improvements are significant. ↑ denotes higher the better and ↓ represents lower the better. The best results are marked in bold.

Methods	Black Background			White Background			Average			*p*-Value
	MAE ↓	PSNR ↑	SSIM ↑	MAE ↓	PSNR ↑	SSIM ↑	MAE ↓	PSNR ↑	SSIM ↑	
GAN	0.080	13.58	0.886	0.074	14.06	0.896	0.077	13.82	0.891	$2.01\times 10^{-29}$
DCGAN	0.040	13.55	0.706	0.031	11.21	0.835	0.035	12.38	0.771	$1.92\times 10^{-20}$
ChromaGAN	0.029	26.5	0.964	0.026	24.56	0.989	0.028	25.53	0.976	$3.74\times 10^{-17}$
WGAN	**0.014**	26.46	**0.989**	0.024	23.94	0.988	0.019	25.20	0.988	$1.77\times 10^{-20}$
WGAN-GP	0.021	24.13	0.985	0.024	23.55	0.987	0.022	23.84	0.986	$5.00\times 10^{-21}$
CycleGAN	**0.014**	26.21	**0.989**	0.025	23.66	0.986	0.019	24.94	0.988	$7.53\times 10^{-12}$
**Ours**	0.019	**27.42**	0.983	**0.015**	**27.97**	**0.995**	**0.017**	**27.70**	**0.989**	-

**Table 2:** Performance comparison between using refined foreground maps and coarse foreground maps. ↑ denotes higher the better and ↓ represents lower the better.

Methods	Black Background			White Background			Average		
	MAE ↓	PSNR ↑	SSIM ↑	MAE ↓	PSNR ↑	SSIM ↑	MAE ↓	PSNR ↑	SSIM ↑
Ours	0.025	21.31	0.972	0.042	19.34	0.970	0.033	20.33	0.971
Ours	0.019	27.42	0.983	0.015	27.97	0.995	0.017	51.81	0.989

**Table 3:** Quantitative results of the ablation study on the cryosection dataset. ↑ denotes higher the better and ↓ represents lower the better.

Methods	Black Background			White Background			Average		
	MAE ↓	PSNR ↑	SSIM ↑	MAE ↓	PSNR ↑	SSIM ↑	MAE ↓	PSNR ↑	SSIM ↑
w/o color	0.040	20.59	0.947	0.049	19.28	0.966	0.045	19.94	0.957
w/o gan1	0.015	27.88	0.987	0.022	25.37	0.994	0.019	26.63	0.991
w/o gan2	0.049	20.97	0.901	0.028	23.50	0.991	0.039	22.24	0.946
Ours	0.019	27.42	0.983	0.015	27.97	0.995	0.017	27.70	0.989

## Data Availability

All the data used in this study are publicly available at 18 November 2022 https://www.nlm.nih.gov/research/visible/visible_human.html.
